# Virtual clinical examinations: are they the new ‘normal'?

**DOI:** 10.1192/bjo.2021.361

**Published:** 2021-06-18

**Authors:** Mahmoud Barakat, Hannah Slevin, Reshmi Nijjar, Kavitha Ramamurthy, Latha Hackett, Dushyanthan Mahadevan

**Affiliations:** 1 Manchester Foundation Trust; 2 East Lancashire Hospitals NHS Trust; 3 Health Education England North West

## Abstract

**Aims:**

The North West School of Psychiatry run a yearly structured clinical examination to help Core Psychiatry Trainees develop their training competencies and prepare for the MRCPsych Clinical Assessment of Skills and Competencies (CASC). Historically, this has been face-to-face with logistical difficulties, high cost, low trainee uptake and challenging in recruiting examiners. Following the COVID-19 pandemic and the subsequent shift to virtual consultations and examinations, the team implemented an innovative virtual Skills test. The main aims were to improve the test's quality and the trainee uptake, adapt the test delivery to a Health Education England (HEE) online platform, and establish cost-effectiveness in the post-COVID world.

**Method:**

A working group was formed to develop the Skills test, and in May 2019, the test was delivered face-to-face, implementing 5 cycles of 8 stations over 3 days. The same group adapted the test for online delivery, and in August 2020, 3 cycles of 8 stations were delivered. Feedback was collected, with adaptations made for a second Skills Test in December 2020.

**Result:**

96.4% of trainees rated their overall experience and the test organisation in the 2019 test as excellent or good (82.1% and 85.7 excellent, respectively). 93.5% of examiners rated their overall experience and the test organisation as excellent or good (45.1% excellent for both). In the August test, 95.8% of trainees rated their overall experience as excellent or good (58.3% excellent). 100% of trainees rated the test organisation and the online format as excellent or good (70.8% and 50% excellent, respectively). Although 100% of examiners rated the overall experience, the test organisation and online format as excellent or good, some felt the stations were not long enough to allow for technical issues. In the December test, higher proportions of trainees rated the overall experience (80%), organisation of the test (80%) and online format (70%) as excellent.

**Conclusion:**

The virtual test is shown to be a viable and successful alternative to the face-to-face test in preparing trainees for their CASC, and trainees felt it was excellent preparation for the new online CASC format. It had some clear advantages, such as saving on consumables, reducing the financial costs of running the test, improving the test quality, and increasing the trainee uptake. It is more eco-friendly and reduces fuel emission, raising the question of how the test should be delivered after the COVID-19 pandemic.

